# Animal derived products may conflict with religious patients’ beliefs

**DOI:** 10.1186/1472-6939-14-48

**Published:** 2013-12-01

**Authors:** Axelina Eriksson, Jakob Burcharth, Jacob Rosenberg

**Affiliations:** 1 Department of Surgery D, Herlev Hospital, University of Copenhagen, Herlev Ringvej 75, Herlev DK2730, Denmark

## Abstract

**Background:**

Implants and drugs with animal and human derived content are widely used in medicine and surgery, but information regarding ingredients is rarely obtainable by health practitioners. A religious perspective concerning the use of animal and human derived drug ingredients has not thoroughly been investigated. The purpose of this study was to clarify which parts of the medical and surgical treatments offered in western world-hospitals that conflicts with believers of major religions.

**Methods:**

Religious and spiritual leaders of the six largest religions worldwide (18 branches) were contacted. A standardised questionnaire was sent out regarding their position on the use of human and animal derived products in medical and surgical treatments.

**Results:**

Of the 18 contacted religious branches, 10 replied representing the 6 largest religions worldwide. Hindus and Sikhs did not approve of the use of bovine or porcine derived products, and Muslims did not accept the use of porcine derived drugs, dressings or implants. Christians (including Jehovah’s Witnesses), Jews and Buddhists accepted the use of all animal and human derived products. However, all religions accepted the use of all these products in case of an emergency and only if alternatives were not available.

**Conclusions:**

The views here suggest that religious codes conflict with some treatment regimens. It is crucial to obtain informed consent from patients for the use of drugs and implants with animal or human derived content. However, information on the origin of ingredients in drugs is not always available to health practitioners.

## Background

Rules and customs regarding consumption and use of specific animals differ among different religious branches. Religious beliefs potentially conflict with specific health care situations in cases of removing a patient from ventilator support (Orthodox Jews), blood transfusions (Jehovah’s Witnesses), fluid and nutrition therapy (Roman Catholics) and ending life in patients with documented brain death (Buddhists) [1-3].

In modern medicine, animal derived products are used in many fields, e.g. anaesthesiology, psychiatry, orthopaedic, plastic and general surgery [3-8], which potentially can create conflicts with religious beliefs. No centralized information is available for either patients or physicians regarding animal derived ingredients in drugs, implants or dressings [5,9-11]. In cases of conflicts between religious beliefs and needed treatment, a risk of non-adherence is present [12]. This problem of animal derived ingredients in drugs has been addressed several times in the literature across specialties [2-6,8,10,12-15].

The purpose of this study was to clarify which major religious beliefs that potentially are in conflict with medical or surgical treatments that contain animal derived products.

## Methods

Religious branches exceeding more than 15 million adherents worldwide were included as potential participants in this study [16] (Table 1). The major Christian branches (Catholicism, Orthodoxies, liberal and conservative Protestantism, African indigenous sects, Pentecostalism, Jehovah’s Witnesses and the Anglican Church), the Muslim branches (Sunni and Shiite), the Hindu branches (Vaishnavism, Shavites, and Neo-hindus) and the Buddhist branches (Mahayana, Theravada, and Lamaism) were pooled for each religion (see Table 1).

**Table 1:** Number of adherents for religious branches exceeding more than 15 million adherents

**Religion**	**Branch**	**Number of adherents**
Christianity	Catholic	1,050,000,000
	Orthodox	240,000,000
	Conservative protestant	200,000,000
	Liberal protestant	150,000,000
	African indigenous sects	110,000,000
	Pentecostal	105,000,000
	Anglican	73,000,000
	Jehova's Witnesses	14,800,000
Islam	Sunni	940,000,000
	Shiite	120,000,000
Hinduism	Vaishnavism	580,000,000
	Shavites	220,000,000
	Neo-hindus	22,000,000
Buddhism	Mahayana	185,000,000
	Theravada	124,000,000
	Lamaism	20,000,000
Sikhism	n/a	23,000,000
Judaism	n/a	15,000,000

A questionnaire was designed and face validated [17]. It consisted of 7 questions concerning the religion’s stand towards whether the use of human, porcine or bovine content in drugs, dressings and implants was allowed for adherents of their respective religion. For each question, an example was stated. Drugs used as examples are shown in Table 2. We found this method the most valid, compared to searching texts and statements. The specific information was either not accessible, or could be interpreted differently, or the validity of these texts were meant for a limited group of people in a specific context. The purpose of the study, was emailed to selected religious leaders in 26 different countries (Denmark, Sweden, Germany, United Kingdom, Hungary, Russia, Italy, Greece, USA, Thailand, Malaysia, Nepal, China, India, Lebanon, Iran, Turkey, Serbia, Poland, Belarus, Bangladesh, Sri Lanka, Egypt, Kenya, Syria and Mongolia), which were discovered through homepages of religious branches in the countries where the religion was dominant. If no answer was received by email the religious leaders were contacted via telephone through their organisation and offered the opportunity to answer the questionnaire orally. It was beyond the scope of our study to investigate the physician’s attitude towards the use of drugs and implants with potential animal derived content. There may be other perspectives, than religious, for a person to object to animal-derived treatments. These groups and their beliefs were not addressed in this study.

**Table 2:** **An arbitrary example of well**-**known drugs with animal or human derived ingredients**, **since the full product information is not obtainable**, **to make a full list**

**Type**	**Examples**	**Porcine**	**Bovine**	**Human**
Drugs	Amoxycillin, omeprazole, warfarin, prednisolone, oxinorm, heparin	X	x	
Dressings	Hydrocolloids, split skin graft	X	x	x
Surgical products/implants	Mesh, bone, orthopaedic spacer, matrix haemostasis	X	x	x

There were no conflicts of interest and there was no need for any ethics committees’ permission, which they confirmed, since it the included subjects only were stating the general position for their branch of religion. AE, JB and JR developed the concept, design and disposition for the study. The questionnaire was developed by AE, JB and JR. AE contacted religious and spiritual leaders and analysed the data with JB and JR. The first manuscript draft was written by AE and revised critically by JB and JR. All authors have read and approved the final manuscript.

The drugs used as examples were arbitrarily selected, to be well known drugs where it was possible find the origin of the contents [18,19]. Information on the chosen drugs with animal derived contents was found through the Danish Medicines Agency [18]. Information on dressings and implants were found in the PubMed database.

## Results

Of the 18 contacted religious branches, 10 replied, representing the 6 largest religions worldwide (see Table 3). The major branch of Hinduism, Vaishnavism, did not permit the use of any drugs, dressings or implants, if they contained porcine or bovine material, since they considered killing of animals and especially the killing of cows, sinful. Sikhs did not approve of any use of animal derived products. However, in emergency or starvation situations, those rules could be waived for both Hindus and Sikhs. Even in routine treatment, if patients were lacking reasonable alternatives drugs, bandages or implants containing animal products could be allowed. All such decisions were left to be decided by the individual.

**Table 3:** **Replies from religious branches**, **indicating which products that are allowed**, **and not**

**Clergy responses (N =** **10**)	**Drugs pigs**	**Drugs cows**	**Dressings pigs**	**Dressings cows**	**Implants pigs**	**Implants cows**	**Implants human**
Christianity (Catholics, Protestants, African indigenous sects and Jehovah’ Witnesses)	Yes	Yes	Yes	Yes	Yes	Yes	Yes^2^
Islam (Sunni and Shiite)	No^1^	Yes	No^1^	Yes	No^1^	Yes	Yes
Hindu (Vaishnavism)	No^1^	No^1^	No^1^	No^1^	No^1^	No^1^	Yes^2^
Buddhism (Theravada)	Yes	Yes	Yes	Yes	Yes	Yes	Yes
Sikhism	No^1^	No^1^	No^1^	No^1^	No^1^	No^1^	Yes
Judaism	Yes	Yes	Yes	Yes	Yes	Yes	Yes^2^

We found that Sunni and Shiite Muslims did not approve of drugs, dressings or implants with porcine content. However, as for Hindus and Sikhs these products were allowed, if no other alternative drug existed and the treatment was considered life prolonging. In an emergency situation the use of these drugs was also approved. Sunni Muslims believed it to be mandatory to use these drugs if no alternative treatment was available, since human life is considered more sacred than the haram (forbidden) use of porcine.

Christians (including Jehovah’s Witnesses), Theravada Buddhists and Jews had no problem with the use of drugs, dressings or implants with animal or human derived contents. Christians (including Jehovah’s Witnesses), Hindus and Sikhs, commented that the use of human derived implants were only allowed if a donor had given consent. Jehovas Witnesses commented that only the use of blood derived products was forbidden.

## Discussion

The basic findings of the study were that among the largest (by number of adherents worldwide) religious branches, several of them had restrictions regarding the use of animal derived medical products. Hindus and Sikhs did not accept the use of bovine or porcine containing products, and Muslims did not accept the use of porcine drugs, dressings or implants. Christians, Jehovah’s Witnesses, Jews and Buddhists accepted the use of all animal or human derived drugs, dressings and implants. Interestingly, all religions accepted the use of animal derived products if there were no alternatives or if they were used in an emergency situation.

It is widely accepted that clinicians must inform patients and get consent for each component within a treatment plan [20]. This ethical aspect is considered so crucial that it has become obliged by law in Denmark [21] how this information should be passed on to the patient, is yet to find out. It could be written in the product information, however at the moment it is not possible for the physician to inform the patient since the information is not accessible [22].

To establish whether a drug has animal origin or content, it is possible to contact the manufacturer or a national medicines agency [23]. However, the origin of the ingredients is not always obvious. Examples of drugs with animal derived excipients are shown in Table 2. It is known that gelatine is of animal (porcine or bovine) origin and it has earlier been shown that 50-80% of all capsules contain gelatine [24]. An example of a widespread sold capsule-drug is the proton pump inhibitor omeprazole (Actavis Group, Iceland; Bluefish, Sweden; BMM Pharma, Sweden; Copyfarm, Denmark; Pensa Pharma, Sweden; Recept Pharma, Sweden; Sandoz, Switzerland; Stada, Germany; and many more) [25]. Twelve out of 14 available omeprazole alternatives on the Danish market alone contain gelatine, which in up to 80% of the cases is derived from pigs [18,24].

The extent of the use of animal derived exipients in drugs has previously been addressed in a study from the United States. They found that 15 out of 41 psychotropic medications contained gelatine [3]. All but one heparin-drugs in the UK originated from animals, and the synthetic alternative was not approved for surgeries other than orthopaedic procedures [23]. Furthermore, measles, mumps and rubella vaccines and tablets containing pancreatic enzymes were of animal origin [23].

The use of the use of biological dressings in the treatment of chronic and acute wounds including burns, was discussed in one previous article [26]. Split skin grafts from a donor and dressings derived from animals can be used instead of allo-transplanted skin. The use of these alternative products avoids the donor-site problem on the patient, and animal derived dressings are easier to acquire. These products may be better in certain circumstances compared with synthetic alternatives [26,27]. Hydrocolloids act by autolysis, rehydrating the wound and thereby promote debridement [5,26]. A study found that a majority of healthcare professionals in the UK did not know the origin of animal derived products in frequently used dressings [5]. The same study found that use of porcine and bovine dressings, in adherents of the Chinese society, Jehovah’s Witnesses, Methodists and Muslims, required informed consent for some reason, not stated. Dressings derived from humans used in adherents to the Anglican Church, Jehovah’s Witnesses, Methodists, Quakers, Roman Catholics and Salvation Army, required informed consent [5].

Implants are surgical products left in the body e.g. heart valves, meshes used for hernia repair, and spacers used in orthopaedic surgery. These products are more thoroughly labelled than drugs. The use of biological meshes provides less risk of infection, foreign body reaction, and are overall better integrated with less postsurgical pain and discomfort compared with their synthetic alternatives [28,29]. The religious aspects on the use of biological meshes in comparison with the religious food restrictions were investigated in a previously published study [6]. Representatives from Judaism, Islam, Buddhism, Hinduism, Scientology and several branches of Christianity had no objections on the use of biological meshes. The Church of Jesus Christ of Latter Days Saints felt the need for informed consent for the use of these types of meshes, and the study also found that religious food restrictions did not translate into restrictions in the surgical field. Another study on biological implants and animal derived drugs used in orthopaedic surgery sought guidelines from religious leaders in Australia [7] and found that Hindus did not accept the use of bovine surgical implants, where Muslims permitted the use of porcine surgical products if all other options had been exhausted. Their results differ somewhat from the results in the current article.

In comparison to the present study, previous studies have not focused on world-wide religions, but the most popular religions in their respective countie. The main focus of earlier published studies was to examine whether followers of religious groups would like information on the origin of products. In this study, it was assumed that informed consent should be obtained for the use of these types of products, but the aim was to investigate where the conflicts may appear. Dietary restrictions were not relevant for us when addressing this matter and therefore it did not bias the selection of religions or its outcome. Furthermore, previous studies have investigated the issue in a narrow perspective, concerning only one speciality, one country or not covering drugs with animal origin. Therefore, it was not possible to compare previous studies to the present.

Alternative drugs, dressings or implants without human or animal derived content exist, however not for all products [4,23]. Knowledge of these alternatives is crucial for health practitioners in order to properly guide their patients of Hindu, Sikh or Muslim faith. Therefore, the health practitioner should both have sufficient knowledge on drug and implant ingredients and religious considerations of the treatment regimens.

A limitation of this study was that individual differences in the interpretation of the religion must be considered. The contacted religious leaders were asked to reply on behalf of their adherents, but that does not translate into all adherents having exact the same religious standpoint as that particular spiritual leader. Thus, some Hindus, Muslims and Sikhs may not have a negative standpoint towards animally derived products. Neither does this study conclude that all Christians, Buddhists and Jews accept all of these products and does not want to be informed about the origin of animal derived origin of drugs, dressings or implants.

## Conclusions

In conclusion, it is necessary to obtain informed consent for the use of animal or human derived products for several religions, since they may oppose to the treatment. Hindus, Sikhs and Muslims, do not approve of some animal derived products if there are other alternatives. However, if there are no alternatives, and if the treatment is life-saving, then all religions approved of all treatment modalities regardless of origin.

1. They accepted the use of all animal and human derived products, in case of emergency and only if other alternatives were not available.

2. Religious leaders commented that the donor must have given informed consent.

## Competing interests

The authors declare that they have no competing interests.

## Authors’ contributions

AE designed the study, closely supervised and advised by JB and JR. AE collected the data and analysed it. All three authors made a detailed disposition for the manuscript draft. AE wrote the draft, where JB and JR edited it before submission. All three authors have read and approved the final manuscript.

## Pre-publication history

The pre-publication history for this paper can be accessed here:

http://www.biomedcentral.com/1472-6939/14/48/prepub
